# AI based automatic measurement of split renal function in [^18^F]PSMA-1007 PET/CT

**DOI:** 10.1186/s41824-025-00254-8

**Published:** 2025-06-16

**Authors:** Kristian Valind, Johannes Ulén, Anni Gålne, Jonas Jögi, David Minarik, Elin Trägårdh

**Affiliations:** 1 Department of Translational Medicine, Wallenberg Centre for Molecular Medicine, Lund University, Malmö, Sweden; 2 Department of Clinical Physiology and Nuclear Medicine, Skåne University Hospital, Lund, Sweden; 3 Department of Clinical Physiology and Nuclear Medicine, Skåne University Hospital, Malmö, Sweden; 4 Eigenvision AB, Malmö, Sweden; 5 Department of Radiation Physics, Skåne University Hospital, Malmö, Sweden

**Keywords:** PSMA, PET, SRF, Renal function, AI, CNN, Segmentation

## Abstract

**Background:**

Prostate-specific membrane antigen (PSMA) is an important target for positron emission tomography (PET) with computed tomography (CT) in prostate cancer. In addition to overexpression in prostate cancer cells, PSMA is expressed in healthy cells in the proximal tubules of the kidneys. Consequently, PSMA PET is being explored for renal functional imaging. Left and right renal uptake of PSMA targeted radiopharmaceuticals have shown strong correlations to split renal function (SRF) as determined by other methods. Manual segmentation of kidneys in PET images is, however, time consuming, making this method of measuring SRF impractical. In this study, we designed, trained and validated an artificial intelligence (AI) model for automatic renal segmentation and measurement of SRF in [^18^F]PSMA-1007 PET images.

**Results:**

Kidneys were segmented in 135 [^18^F]PSMA-1007 PET/CT studies used to train the AI model. The model was evaluated in 40 test studies. Left renal function percentage (LRF%) measurements ranged from 40 to 67%. Spearman correlation coefficients for LRF% measurements ranged between 0.98 and 0.99 when comparing segmentations made by 3 human readers and the AI model. The largest LRF% difference between any measurements in a single case was 3 percentage points. The AI model produced measurements similar to those of human readers.

**Conclusions:**

Automatic measurement of SRF in PSMA PET is feasible. A potential use could be to provide additional data in investigation of renal functional impairment in patients treated for prostate cancer.

**Supplementary Information:**

The online version contains supplementary material available at 10.1186/s41824-025-00254-8.

## Background

Positron emission tomography (PET), combined with computed tomography (CT) into hybrid PET/CT studies, plays an important part in the diagnosis and staging of many malignancies, as well as in detection and localization of recurrence. For prostate cancer, the transmembrane protein glutamate carboxypeptidase II, also called prostate-specific membrane antigen (PSMA), has become the main molecular imaging target. PSMA PET is used for initial staging of patients with high-risk prostate cancer, localization of biochemical recurrence, and for evaluation of tumor PSMA expression prior to radioligand therapy (Fendler et al. 2023). Overexpression of PSMA in prostate cancer enables PET imaging and targeted therapy. However, PSMA is also physiologically expressed in the proximal tubules of the kidneys, in addition to other normal tissues (Silver et al. 1997; Kinoshita et al. 2006). Several radiopharmaceuticals targeting PSMA are being investigated for functional imaging of the kidneys (Sarikaya et al. 2018; Schierz et al. 2021; Rassek et al. 2023; Valind et al. 2023a). Such use could potentially be relevant as urinary obstruction—a known complication after surgical or radiological treatment for prostate cancer, which may lead to impaired renal function (Heemsbergen et al. 2010).

The left–right uptake distributions of [^68^Ga]Ga-PSMA-11 and [^18^F]PSMA-1007 in the kidneys have been shown to correlate significantly to split renal function (SRF) as measured with technetium-99m labelled mercaptotriglycene ([^99m^Tc]Tc-MAG3) renography and dimercaptosuccinic acid ([^99m^Tc]Tc-DMSA) scintigraphy respectively (Rosar et al. 2020; Valind et al. 2023b). Thus, measuring SRF in PSMA PET studies is possible. However, manual segmentation of kidneys in PET images is time-consuming. In this study, we developed, trained and evaluated an artificial intelligence (AI) model for automatic renal segmentation and measurement of SRF in [^18^F]PSMA-1007 PET/CT.

## Methods

### Patient exams

200 [^18^F]PSMA-1007 PET/CT exams, of which 100 included contrast enhanced CT (ceCT) of diagnostic quality, and 100 instead included low-dose CT (ldCT), were randomly selected from a pool of exams where prostate tumors and metastases had been segmented for other studies (Trägårdh et al. 2022; Hvittfeldt et al. 2022). All exams in the pool were clinical [^18^F]PSMA-1007 PET/CT performed at the Department of Department of Clinical Physiology and Nuclear Medicine, Skåne University Hospital, Lund or Malmö, Sweden. All patients had provided written informed consent.

Using random selection 120 of these 200 exams were designated for training, 40 for validation and 40 as a test group. For all these subgroups, the ratio of ldCT to ceCT was kept at 1:1. During the initial validation stage, we found that the model performed poorly on studies with low or markedly asymmetric renal uptake. To remedy this, 15 additional cases with one or both of these characteristics were identified, segmented and added to the training data, bringing the size of the training set to 135 cases. The additional cases consisted of 6 cases with only one kidney each, 5 with one hypoplastic kidney each, 1 with symmetric low uptake, and 4 with asymmetric low uptake).

59% of the 215 studies had been performed for staging of high-risk disease, and 41% for localization of recurrence. The subjects of the studies were aged 71 ± 7 years (mean ± standard deviation), with a weight of 87 ± 15 kg and height of 178 ± 7 cm. There were no significant differences between the training group and the test group regarding these parameters.

### Imaging

All PET images were acquired on a GE Discovery MI (GE Healthcare, Milwaukee, WI, USA) 2 h after injection of 4 MBq/kg of [^18^F]PSMA-1007 (ABX, Radeberg, Germany). For image reconstruction, a block-sequential regularization expectation maximization algorithm (Q.Clear; GE Healthcare, Milwaukee, WI, USA) had been used, with a beta value of 800 (Trägårdh et al. 2020a). Images had been reconstructed with point spread function modelling, time of flight, 256 × 256 matrix size and 2.73 × 2.73x2.79 mm voxel size.

### Segmentation

One nuclear medicine physician (KV, reader 1) performed segmentation of left and right kidneys in all PET images, using the RECOMIA (Trägårdh et al. 2020b) platform. In addition to reader 1, the test group was also segmented by two additional nuclear medicine physicians (AG and ET). All readers performed segmentation with the PET images displayed with a standardized uptake value (SUV) scale from 0 to the approximate maximum SUV of each kidney.

### AI model

The AI model was based on a 3D U-Net architecture (Çiçek et al. 2016). The input to the model was both the CT and SUV image resampled to the same pixel size, 2.73 × 2.73x2.79 mm. The output was a pixelwise score for each pixel for background, left kidney and right kidney. The AI model was trained and evaluated with same padding convolutions with patches of 160 × 160x160 pixels. Due to the limited amount of data, data augmentation was used to increase the diversity of the training data by creating new patches from existing ones. The images were scaled, translated, sheared, and rotated randomly. The Hounsfield unit (HU) values were also randomly changed, smoothed, and random gaussian noise was added. The model was trained using categorical cross-entropy loss, which measures the prediction error of the model, and deep supervision (Lee et al. 2015), which add loss function on every scale of the model. The loss function was weighted with the kidney pixels given a weight of 5 and background a weight of 1.

The AI model was optimized using the ADAM (Kingma and Adam 2014) optimizer with Nesterov momentum. The learning rate controls how much each example update the weights, it was initialized to 0.00005 and reduced 1.5% each epoch. Each epoch consisted of 10,000 random samples and the model was trained for a total of 100 epochs. As a postprocessing step only the largest region, defined as the largest connected component (26-connected) was kept for left and right kidney.

### Statistical analysis

All human and AI model segmentations were compared against each other. All human segmentations thus in turn served as the ground truth for comparing the other human and AI segmentations. The volume of each renal segmentation and the left renal function percentage (LRF%) was measured and compared to the ground truth. LRF% was calculated by summing the SUV of all voxels in the left kidney and then dividing by the summed voxels of both left and right kidneys. In each segmentation comparison, Dice-Sørensen coefficient (DSC) and difference between segmentation volume were calculated for each individual kidney, as well as the difference in LRF% for each case. Spearman correlation coefficients were calculated for LRF% and renal volume measurements. Bland–Altman analysis was performed for the LRF% measurements. Two-tailed T-tests were used to test for significant differences between the training and test groups regarding participant characteristics (age, height and weight).

## Results

**Fig. 1:**
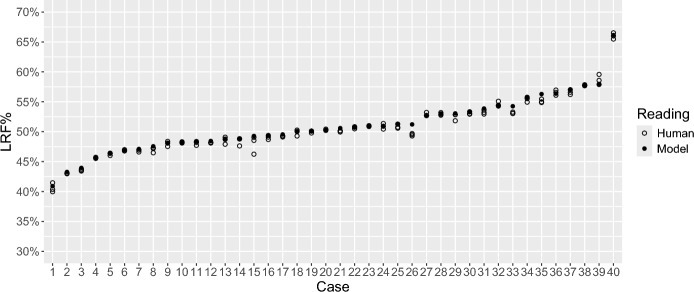
LRF% measurements. LRF% measurements from human (white circles) and model (black circles) segmentations, ordered from lowest to highest. LRF%, left renal function percentage

**Fig. 2:**
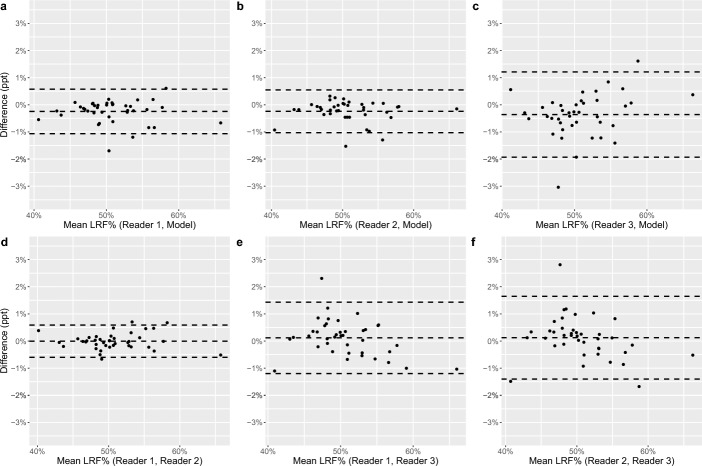
Bland–Altman plots comparing LRF% measurements. In **a**, **b**, and **c** the AI model is compared against readers 1, 2 and 3 respectively. In d and e readers 2 and 3 are compared to reader 1. In f, reader 3 is compared to reader 2. The upper and lower dashed lines represent 95% confidence intervals. The central dashed line represents the mean difference. LRF%, left renal function percentage; ppt, percentage points

LRF% measurements in the test dataset ranged from 40 to 67% (Fig. 1, Supplement 1). This includes all measurements by all human readers and the model. The largest difference of LRF% measurements between all readings (human and model) in any individual was 3 percentage points (ppt) (Table 1). Spearman correlation coefficients for LRF% ranged from 0.98 to 0.99, with similar values for human-to-human and model-to-human comparisons. Bland–Altman (BA) analysis for all comparisons yielded limits of agreement at or below 3 ppt, and bias of -0.2, -0.2 and -0.4 when the model was compared to readers 1, 2 and 3 respectively. The bias was and up to 0.1 ppt when comparing the human readers with each other. BA plots comparing LRF% measurements are presented in Fig. 2, with correlation plots in Supplement 2. Renal volume measurements ranged from 121 to 530 ml, with the largest difference between measurements of the same kidney being 127 ml (Table 1). Spearman correlation coefficients for renal volume measurements ranged from 0.93 to 0.98.

**Table 1:** Volume difference in milliliters, LRF% difference in percentage points and Dice-Sørensen comparisons between segmentations. Data presented as median (min–max). DSC, Dice-Sørensen coefficient; Diff_vol_, volume difference; Diff_LRF%_, difference in left renal function percentage

Reader 1 vs Model	Reader 2 vs Model	Reader 3 vs Model
Diff_vol_: − 0.6 (− 30.9 to 35.9) DSC: 0.96 (0.90 to 0.99) Diff_LRF%_: − 0.2 (− 1,7to 0.6)	Diff_vol_: 15.7 (19.0 to 63.6) DSC: 0.95 (0.89 to 0.99) Diff_LRF%_: − 0.2 (− 1,5 to 0.3)	Diff_vol_: − 33.3 (− 99.8 to − 5.2) DSC: 0.92 (0.81 to 0.97) Diff_LRF%_: − 0.3 (− 3.0 to 1.6)

Reader 1 vs Reader 2	Reader 1 vs Reader 3	Reader 2 vs Reader 3
Diff_vol_: − 17.1 (− 51.8 to 23.1) DSC: 0.95 (0.89 to 0.99) Diff_LRF%_: 0 (− 0.7 to 0.7)	Diff_vol_: 32.4 (0.3 to 98.8) DSC: 0.92 (0.78 to 0.99) Diff_LRF%_: 0.2 (− 1.1 to 2.3)	Diff_vol_: 46.9 (19.0 to 126.5) DSC: 0.89 (0.75 to 0.97) Diff_LRF%_: 0.2 (− 1.7 to 2.8)

## Discussion

In this study, we developed, trained and evaluated an AI model for automatic segmentation of kidneys and measurement of SRF in [^18^F]PSMA-1007 PET. We found that our model produced measurements similar to those of human readers. Thus, automatic measurement of SRF is feasible in [^18^F]PSMA-1007 PET. This could allow using SRF data from a PSMA PET/CT, performed as part of initial prostate cancer staging, as baseline when investigating suspect renal impairment related to urinary obstruction, e.g. with [^99m^Tc]Tc-MAG3 renography. Urinary obstruction is a known complication to radiotherapy or surgical treatment of prostate cancer, appearing in around 5% of treated patients within 3 years (Heemsbergen et al. 2010). It should be noted, however, that SRF from [^18^F]PSMA-1007 PET does not provide a complete picture of the patient’s renal function and does not replace other renal function parameters such as glomerular filtration rate (GFR). Furthermore, measurement of SRF is not always necessary in management of impaired renal function as a complication to treatment of prostate cancer.

We have previously compared manual measurements of SRF using [^18^F]PSMA-1007 PET and [^99m^Tc]Tc-DMSA single photon emission computed tomography (SPECT) and found a strong correlation (Pearson’s r = 0.99) between the modalities (Valind et al. 2023b). Rosar and coauthors compared SRF between [^68^Ga]Ga-PSMA-11 and [^99m^Tc]Tc-MAG3 renography with a similarly strong correlation (Pearson’s r = 0.91) (Rosar et al. 2020).

The present study however is the first that we are aware of that evaluates automatic measurement of SRF in PSMA PET.

The LRF% measurements based on the AI model segmentations had a slight negative bias (a maximum of -0.4 ppt), which lacks clinical significance. The AI model segmentations were otherwise similar to those by reader 1 with a median DSC of 0.96 (Table 1), which is expected as reader 1 performed segmentation of the training data. Comparisons against reader 2 and 3 resulted in slightly lower median DSCs of 0.95 and 0.92 respectively. One reason for this relatively high degree of concurrence could be that detailed segmentation instructions were provided to the readers. Using less detailed instructions would probably have resulted in lower DSCs and larger differences in volume measurements between the model and the readers. However, LRF% measurements would probably have been similar as long as the human segmentations were done with a consistent method for each exam.

**Fig. 3:**
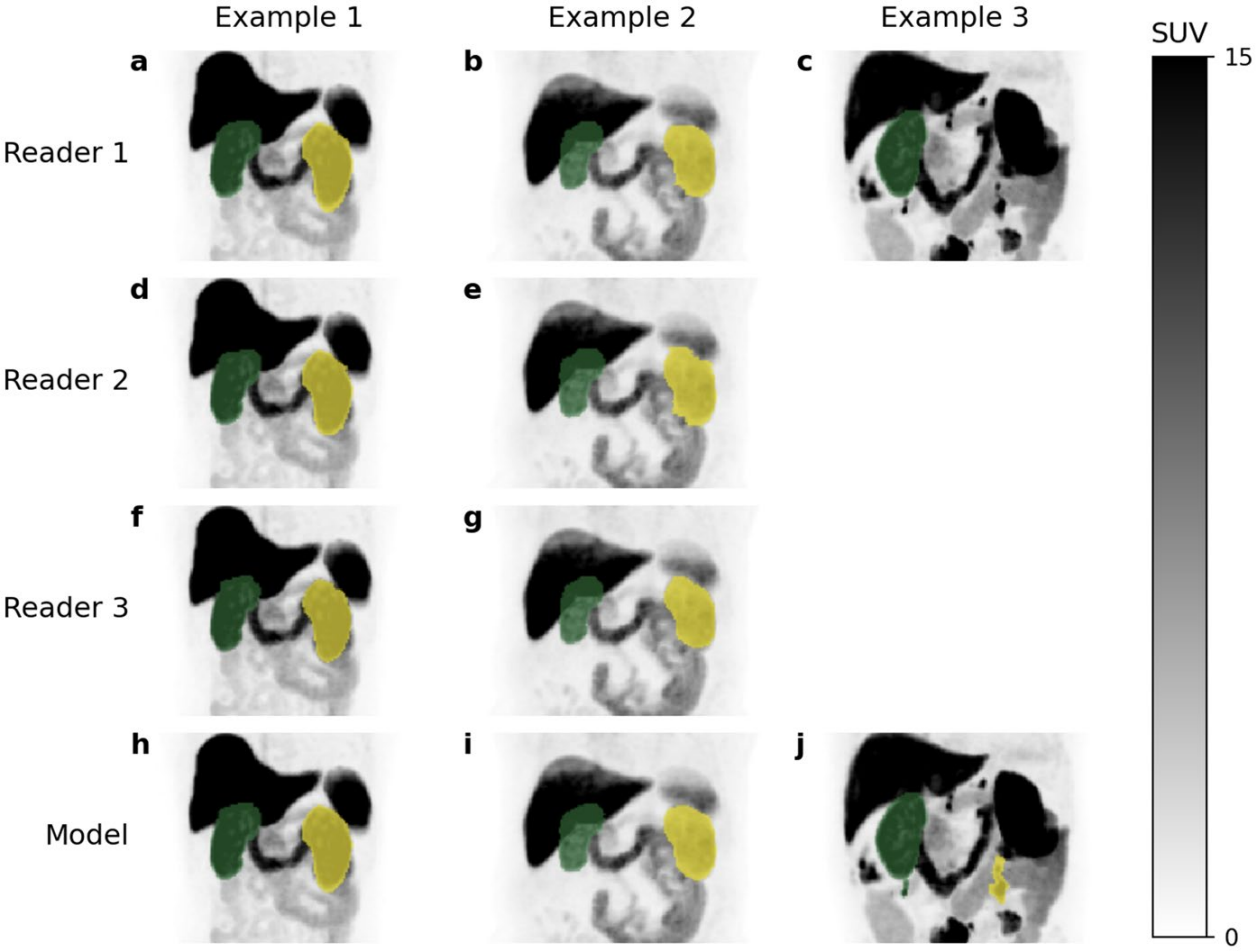
Example segmentations by AI model (h–j) and human readers 1–3. (**a**–**c**, **d**–**e**, **f**–**g** respectively). Example 1 (a, d, f, h) and 2 (b, e, g, i) are from the test dataset. Example 3 is from the validation set and only segmented by reader 1 and the model. SUV, standardized uptake value

This study is not without limitations. First, the limited range of measured LRF% in the test set (40–67%) means our model has not been thoroughly evaluated for subjects with markedly asymmetric renal function. As shown in (j) in Fig. 3, the AI model erroneously segmented lymph node metastases as the left kidney in a case with only a right kidney. This exam was part of the validation set, meaning this error did not affect the measured performance of the AI model, as only the test set was used for evaluation. Adding 15 additional cases with low and/or asymmetric uptake did not significantly improve the AI model’s segmentation of this exam. It is likely that the AI model will have similar suboptimal performance in other cases of markedly asymmetric renal function. Anatomic variants absent from the training set, such as horseshoe kidney, will likely not be handled correctly by the AI model. A larger, more diverse training set, preferably by at least an order of magnitude, could improve the model’s accuracy in cases with less common kidney variants. Finally, the clinical utility of automatic SRF measurement from PSMA PET studies remains somewhat unclear.

## Conclusion

Our AI model for renal segmentation and measurement of split function in [^18^F]PSMA-1007 PET/CT performs similarly to human readers, in cases without markedly asymmetric renal function. This demonstrates the feasibility of automatic measurement of SRF in PSMA PET/CT, which could provide additional data in investigation of renal functional impairment in patients treated for prostate cancer.

## Supplementary Information

Supplementary material 1 (DOCX 24 KB)

Supplementary material 2 (DOCX 66 KB)

## Data Availability

The datasets used for study are available from the corresponding author on reasonable request and relevant ethical approval.

## Abbreviations

AI: Artificial intelligence
ceCT: Contrast enhanced computed tomography
CNN: Convolutional neural network
CT: Computed tomography
DMSA: Dimercaptosuccinic acid
DSC: Dice-Sørensen coefficient
GFR: Glomerular filtration rate
HU: Hounsfield unit
ldCT: Low-dose computed tomography
LRF%: Left renal functional percentage
MAG3: Mercaptotriglycene
PET: Positron emission tomography
ppt: Percentage point
PSMA: Prostate-specific membrane antigen
SPECT: Single photon emission computed tomography
SRF: Split renal function
SUV: Standardized uptake value
