# Supplementary material for: AI based automatic measurement of split renal function in [18F]PSMA-1007 PET/CT
Source: EJNMMI Rep. 2025 Jun 16;9(1):20. doi: 10.1186/s41824-025-00254-8 (PMC12167725; doi:10.1186/s41824-025-00254-8)
Supplement: Supplementary file 1 — Supplementary material 1 (DOCX 24 KB) [file 41824_2025_254_MOESM1_ESM.docx]

Supplement 1. Table of LRF% measurements, with per-case difference between smallest and largest measurement per. LRF% = left renal function percentage. ppt = percentage points

| Case | Reader 1 | Reader 2 | Reader 3 | AI model | Max difference (ppt) |
| --- | --- | --- | --- | --- | --- |
| 1 | 40% | 40% | 41% | 41% | 1% |
| 2 | 43% | 43% | 43% | 43% | 0% |
| 3 | 44% | 44% | 43% | 44% | 1% |
| 4 | 46% | 46% | 46% | 46% | 0% |
| 5 | 46% | 46% | 46% | 46% | 0% |
| 6 | 47% | 47% | 47% | 47% | 0% |
| 7 | 47% | 47% | 47% | 47% | 1% |
| 8 | 47% | 47% | 46% | 48% | 1% |
| 9 | 48% | 48% | 48% | 48% | 1% |
| 10 | 48% | 48% | 48% | 48% | 0% |
| 11 | 48% | 48% | 48% | 48% | 1% |
| 12 | 48% | 48% | 48% | 48% | 0% |
| 13 | 49% | 49% | 48% | 49% | 1% |
| 14 | 49% | 49% | 48% | 49% | 1% |
| 15 | 49% | 49% | 46% | 49% | 3% |
| 16 | 49% | 49% | 49% | 49% | 1% |
| 17 | 49% | 49% | 49% | 50% | 0% |
| 18 | 50% | 50% | 49% | 50% | 1% |
| 19 | 50% | 50% | 50% | 50% | 0% |
| 20 | 50% | 50% | 50% | 50% | 0% |
| 21 | 50% | 50% | 50% | 51% | 1% |
| 22 | 51% | 51% | 50% | 51% | 0% |
| 23 | 51% | 51% | 51% | 51% | 0% |
| 24 | 51% | 50% | 51% | 51% | 1% |
| 25 | 51% | 51% | 51% | 51% | 1% |
| 26 | 50% | 50% | 49% | 51% | 2% |
| 27 | 53% | 53% | 53% | 53% | 1% |
| 28 | 53% | 53% | 53% | 53% | 0% |
| 29 | 53% | 53% | 52% | 53% | 1% |
| 30 | 53% | 53% | 53% | 53% | 0% |
| 31 | 54% | 53% | 53% | 54% | 1% |
| 32 | 54% | 54% | 55% | 54% | 1% |
| 33 | 53% | 53% | 53% | 54% | 1% |
| 34 | 56% | 56% | 55% | 56% | 1% |
| 35 | 55% | 55% | 55% | 56% | 1% |
| 36 | 57% | 56% | 57% | 56% | 1% |
| 37 | 56% | 57% | 57% | 57% | 1% |
| 38 | 58% | 58% | 58% | 58% | 0% |
| 39 | 59% | 58% | 60% | 58% | 2% |
| 40 | 65% | 66% | 67% | 66% | 1% |
