# Supplementary material for: AI based automatic measurement of split renal function in [18F]PSMA-1007 PET/CT
Source: EJNMMI Rep. 2025 Jun 16;9(1):20. doi: 10.1186/s41824-025-00254-8 (PMC12167725; doi:10.1186/s41824-025-00254-8)
Supplement: Supplementary file 2 — Supplementary material 2 (DOCX 66 KB) [file 41824_2025_254_MOESM2_ESM.docx]

Supplement 2. Correlation plots comparing LRF% measurements. In a, b, and c the AI model is compared against readers 1, 2 and 3 respectively. In d and e readers 2 and 3 are compared to reader 1. In f, reader 3 is compared to reader 2. The r values represent Spearman correlation coefficients. LRF% = left renal function percentage.
